# Post-Receptor Crosstalk between Growth Hormone and Insulin Signal in Rats Born Small for Gestational Age with Catch-Up Growth

**DOI:** 10.1371/journal.pone.0100459

**Published:** 2014-06-25

**Authors:** Hong-Zhu Deng, Hong Deng, Chao-Qun Cen, Kai-Yun Chen, Min-Lian Du

**Affiliations:** 1 Department of Pediatrics, the Third Affiliated Hospital of Sun Yat-sen University, Guangzhou, China; 2 Department of Infectious diseases, the Third Affiliated Hospital of Sun Yat-sen University, Guangzhou, China; 3 Department of Pediatrics, the First Affiliated Hospital of Sun Yat-sen University, Guangzhou, China; Virgen Macarena University Hospital, School of Medicine, University of Seville, Spain

## Abstract

**Objective:**

Insulin resistance has been observed in individuals born small for gestational age (SGA) with catch-up growth (CUG), yet the mechanisms involved remain unclear. This study examined the role of GH and insulin signaling crosstalk in insulin resistance of SGA rats with CUG.

**Design and Methods:**

SGA rats were developed by dietary restriction in pregnant rats. GH receptor inhibition was performed on four-week old CUG-SGA and AGA rats. Phosphorylation of IRS-1, AKT, and ERK, and expression of SOCS3 in the skeletal muscle were determined via immunoblot analysis at baseline and after insulin stimulation in CUG-SGA, NCUG-SGA and AGA groups.

**Results:**

Compared to AGA controls, phosphorylation of IRS-1 and AKT in response to insulin stimulation in CUG-SGA rats was significantly blunted (*P*<0.05), and phosphorylation of ERK at baseline was dramatically activated (*P*<0.05). SOCS3 expression was significantly increased in CUG-SGA compared to AGA (*P* = 0.001) and NCUG-SGA (*P* = 0.006) rats, and was significantly suppressed following *GHR* inhibition (*P*<0.05). Furthermore, phosphorylation of IRS-1 and AKT in response to insulin stimulation increased after *GHR* inhibition (*P*<0.05).

**Conclusions:**

Insulin resistance in CUG-SGA rats is associated with impairment of IRS-1-PI3K-AKT signaling, which may result from GH signaling-induced up-regulation of SOCS3.

## Introduction

Accumulating evidence demonstrates that metabolic syndrome and associated obesity, dyslipidemia, hypertension, insulin resistance and diabetes in adults closely correlate with being born small for gestational age (SGA) [1]–[5]. Approximately 80% of the children born SGA demonstrate catch-up growth (CUG), which generally occurs in the first few years of postnatal life. Previous studies indicated that insulin resistance in children born SGA is associated with height and weight CUG [6]. The molecular mechanisms of insulin resistance in children born SGA with CUG, however, are still poorly understood.

The elevated levels of the growth hormone (GH)/insulin-like growth factor (IGF-1) axis had been found in SGA rats with CUG in our previous study [7]. GH promotes longitudinal growth and somatic maturation in children and adolescents and is also an important regulator of substrate metabolism and insulin sensitivity. In the post-absorptive phase, where endogenous GH secretion is stimulated, GH promotes lipolysis and oxidation of fatty acids at the expense of glucose [8]. The predominant GH signaling cascade comprises activation of the growth hormone receptor (*GHR*) dimer, phosphorylation of Janus kinase 2 (JAK2) and subsequently of signal transductor and activator of transcription 5 (STAT5). Insulin is also a key hormone regulating metabolism and growth. Insulin binding to the insulin receptor (*IR*) results in phosphorylation/activation of the *IR*, and activates the phosphatidylinositol 3-kinase (PI3K)/Akt and the extracellular signal-regulated kinase 1/2(ERK1/2) pathways. Insulin-stimulated glucose transport into skeletal muscle depends on the activation of a signaling cascade involving insulin receptor substrate 1 (IRS-1), PI3K and Akt. Due to their important roles in growth and metabolism, GH and insulin can functionally interact with each other, regulating cellular metabolism. In addition, recent animal and *in vitro* evidence suggest that GH and insulin can share post-receptor signaling pathways, and these pathways may contribute to GH-induced insulin resistance [9]. Convergence has been reported at the level of suppressor of cytokine signaling 3 (SOCS3) as well as on protein kinases comprising the insulin signaling pathway, including insulin receptor substrate-1/2 (IRS1/2), PI3K, Akt, and ERK1/2 [10], [11]. However, the interaction by signaling cross-talk between GH and insulin signaling pathways has not been confirmed in SGA models *in vivo*. This may relate to the design of these studies.

Both GH resistance and insulin resistance are present in SGA subjects, which regulated by GH and insulin signals in metabolism and growth. Hence, we hypothesize that the post-receptor crosstalk of GH and insulin signaling would have an effect on CUG and insulin resistance in SGA. In the present study, we examine the insulin resistance in skeletal muscle tissue obtained from animals born SGA with CUG. We aim to confirm whether inhibiting *GHR* under baseline conditions can alter the post-receptor activity of GH and insulin signaling, and explore the possible mechanisms linking CUG and insulin resistance in SGA.

## Methods and Procedures

### Reagents

The sources of materials are as follows: short-acting insulin (Novolin, 400 IU/10 ml) produced by Novo Nordisk, Denmark, the *GHR* antagonist Somavert(Pegvisomant,10 mg/10 ml) from Pfizer Inc., USA, and the insulin radioimmunoassay from Pharmacia & Upjohn Diagnostics AB, Sweden. The following primary antibodies were purchased from Cell Signaling, USA: rabbit anti-rat IRS, anti-p-IRS, anti-AKT, anti-p-AKT (Ser473), anti-ERK, anti-p-ERK1/2 (Thr202/Tyr204) and anti-SOCS3 (L210). Rabbit anti-phosphorylation polyclonal antibody was purchased from Abcam, UK.

### Animals and experimental assignment

All animal procedures conformed to the requirements of the Animal Welfare Act, and protocols were approved by the Institutional Animal Care and Use Committee of Sun Yat-sen University. The animals were housed under conditions approved by the Association for the Assessment and Accreditation of Laboratory Animal Care International.

A total of forty-five healthy female and twenty healthy male Sprague-Dawley rats weighing 230 to 280 g [animal license No. SYXK (Guangdong Province, China) 2007-0081] were obtained from the Animal Center, North Campus of Sun Yat-sen University in China. Standard rat fodder [license No. SCXK (Guangdong Province, China) 2003-0002; Guangdong supervison No. 2008D002] was provided by the Medical Laboratory Animal Center of Guangdong Province in China. Pregnant rats subjected to dietary restriction were utilized to establish the SGA rat model [17]. Rats were randomly housed in standard rat cages at a 2∶1 female-to-male ratio. A vaginal smear examination under a standard optical microscope was performed daily, and the day on which sperm appeared in the smear was determined as day 1 (D1) of pregnancy.

Pregnant female rats were randomly divided into either the dietary restriction or the control group. Rats in the control group were fed 18–20 g/d, whereas rats in the dietary restriction group were fed only 8–9 g/d (approximately 50% of normal intake) after D1.

### Offspring

All pups were weighed at birth and litter sizes were recorded. Litter size was adjusted to 8–10 pups per litter, with random pups excluded if the litter was over 10 pups in size. The pups of each litter in the SGA group were marked with 3% methyl violet after delivery and with 3% picric acid after two weeks. Post-delivery, the mother rats in the two groups were fed a standard control diet. The pups were fed by breastfeeding for the first 3 weeks, and thereafter they were housed in separate cages and fed the same diet as their mothers until the completion of the experiment. Food intakes of the maternal and young rats at 4 weeks of age in the different groups are shown in Table 1. No obvious difference in the food intakes was observed among the CUG-SGA, NCUG-SGA and AGA groups (Table 1). The weight and nose-anus length of all pups were recorded weekly.

**Table 1:** Food intakes of the maternal and young rats in different groups.

		Food intake, g/day	
	AGA	NCUG-SGA	CUG-SGA
Maternal rats	20.97±3.14	18.36±2.23	20.08±2.52
Young rats at 4 weeks of age			
Males(n = 31,36,and 34)	9.43±1.95	8.53±2.17	10.04±2.33
Females(n = 25,33,and 20)	8.75±1.81	8.24±1.96	9.27±2.15

The numbers of rats in the maternal appropriate for gestational age (AGA), small for gestational age with catch-up growth (CUG-SGA) and small for gestational age with no catch-up growth (NCUG-SGA) groups were 15, 20 and 20, respectively. Young rats at 4 weeks: n = 56 in AGA group, n = 54 in CUG-SGA group, n = 69 in NCUG-SGA group. There are not significant differences in AGA, CUG-SGA and NCUG-SGA groups. Food intakes of young male rats in different groups were comparable to those of females respectively.

### Animal model of CUG

SGA infant rats were defined as having a birth weight and/or body length greater than two standard deviations (SDs) below the average birth weight and/or body length of the control group. Non-CUG-SGA (NCUG-SGA) animals were defined by a body weight and body length greater than two SDs below the average body weight and body length of the control group at four weeks of age. Criteria for CUG-SGA rats consisted of body weights and body lengths less than two SDs below the average body weight and body length of the control group at four weeks of age.

### Insulin stimulation

According to body weight and length, all four-week old offspring rats in the three groups [CUG-SGA, NCUG-SGA and appropriate for gestational age (AGA)] were further separated into two sub-groups, namely control and insulin stimulation groups. The insulin stimulation group animals received intraperitoneal injection of 3.0 IU/100 g insulin (diluted to 2 mL with 0.9% saline) 30 min before sacrifice. Control group animals were given intraperitoneal injection of 2 mL saline 30 min prior to sacrifice.

### *GHR* inhibition

Four-week old rats in either the CUG-SGA or AGA groups were further divided into three sub-groups, including control, insulin stimulation and insulin stimulation plus *GHR* inhibition. Control group animals were given intraperitoneal injection of 2 mL saline at 50 hr, 2 hr, and 30 min before sacrifice. Insulin stimulation group animals were administrated intraperitoneal injection of saline at 50 hr and 2 hr pre-sacrifice, followed by injection of 3.0 IU/100 g insulin 30 min before sacrifice. The insulin stimulation plus *GHR* inhibition group animals received 0.001 mg/g Somavert via intraperitoneal injection at 50 hr and 2 hr pre-sacrifice, followed by an injection of 3.0 IU/100 g insulin 30 min before sacrifice.

### Measurements of biochemical parameters

Animals in each group were fasted for 12 hr and sacrificed by 10% chloralic hydras (0.3 mg/kg) injection. Blood and gastrocnemius specimens were extracted and stored at −70°C until laboratory assessment. Serum glucose was determined by a peroxidase method (Photometric Instrument 4010, Roche, Switzerland) (between-group variance <2.0%), and serum insulin was measured using an immunoradiometric assay (Pharmacia & Upjohn Diagnostics AB, Sweden) (between-group variance <5.3%, within-group variance <7.6%). Homoeostasis Model Assessment for Insulin Resistance (HOMA-IR) was used to estimate insulin resistance according to the formula: fasting plasma insulin (µU/mL)*fasting plasma glucose (mmol/L)/22.5 [12].

### Immunoblot analysis

A total of 100 mg of gastrocnemius muscle tissue was extracted from each group and rinsed with PBS 3 times, then homogenized in 1 unit pre-chilled histiocyte lysate consisting of 1 ml LysisBuffer, 5 µl phosphatase, 1 µl protease inhibitors, and 5 µl PMSF. The homogenates were centrifuged at 2000 rpm for 10 min at 4°C. The protein concentrations were measured using the BCA method. Aliquots of protein samples were mixed with an equal volume of 2×SDS buffer solution and heated at 100°C for 5–10 min. Total protein 50 µg/well was resolved and subjected to sodium dodecyl sulfate-polyacrylimide gel electrophoresis (10% gels), and then proteins were electroblotted onto PVDF membranes. The membrane was blocked with 5% bovine serum albumin for 1 h at room temperature and subsequently incubated with primary antibody overnight at 4°C. After being washed in 1×TBST [50 mM Tris/HCl (pH 7.4), 150 mM NaCl and 0.2% Tween-20], the membranes were incubated with a horseradish peroxidase-conjugated anti-rabbit secondary IgG antibody (1: 1,000 dilution in 5%BSA). Signal was detected using an ECL kit (Pierce Biotechnology, USA) and quantified using the Bio-Rad GS-800 scanner (USA). Phosphoprotein content was determined relative to the total protein content (ratio p/T).

### Statistical analysis

Data were analyzed using the Statistical Package for the Social Sciences (SPSS) 13.0 (SPSS Inc., USA). Insulin levels and HOMA-IR were not normally distributed; thus, these values were converted to logarithms and were presented as the geometrical mean ± standard error of the mean (SEM). Other data were presented as the arithmetic mean ± SD. Differences among groups were evaluated by analysis of variance (ANOVA) and interactions were analyzed by least significant difference (LSD). A *P* value<0.05 (two-tailed) was considered statistically significant.

## Results

A total of 249 pups were born in the dietary restriction group, including 224 SGA pups (89.96%). One-hundred-and-twenty-three newborn SGA rats were recruited into the present study. Fifty-four (43.9%) of the 123 SGA rats demonstrated CUG with body weights and lengths less than 2 SDs below those of age- and sex-matched AGA controls at four weeks of age.

### Physical characteristics of the offspring animals

Birth body weights and lengths of CUG-SGA and NCUG-SGA newborn rats were significantly lower than AGA animals (Table 2, *P*<0.05). Body weights and lengths of CUG-SGA rats demonstrated CUG as early as the first week post-delivery, and reached comparable measurements to those of the AGA group by the fourth week. Body weights and body lengths of the NCUG-SGA rats remained significantly lower than those in the AGA group by the fourth week, however (Table 2, *P*<0.05).

**Table 2:** Anthropometric parameters of SGA and AGA groups.

		Body length(cm)			Body weight(g)	
	AGA	CUG-SGA	NCUG-SGA	AGA	CUG-SGA	NCUG-SGA
	(n = 56)	(n = 54)	(n = 69)	(n = 56)	(n = 54)	(n = 69)
**0W**	5.18±0.18	4.57±0.17*	4.41±0.15*	6.28±0.51	4.72±0.37*	4.67±0.35*
**1W**	6.80±0.15	6.66±0.35	6.20±0.46*^#^	14.56±0.58	14.72±2.06	12.93±2.51*^#^
**2W**	8.91±0.37	8.78±0.47	8.11±0.72*^#^	25.68±1.68	26.96±3.04	21.33±4.65*^#^
**3W**	10.67±0.29	10.71±0.50	9.50±0.53*^#^	39.96±2.91	43.93±6.56	32.55±4.62*^#^
**4W**	13.89±0.58	13.62±0.65	12.51±0.58*^#^	72.79±9.43	76.21±4.72	64.98±6.68*^#^

The ratioes of male to female in appropriate for gestational age (AGA), small for gestational age with catch-up growth (CUG-SGA) and small for gestational age with no catch-up growth (NCUG-SGA) groups were 26/30, 29/25 and 41/28. No significant differences were observed in the ratio of male to female among the three groups (p>0.05). *P<0.05 CUG-SGA or NCUG-SGA *vs* AGA; ^#^ *P*<0.05 CUG-SGA *vs* NCUG-SGA; Data are means±SD.

### Insulin resistance index

No significant differences were observed in the levels of serum glucose among the three groups. The serum insulin and HOMA-IR values in the CUG-SGA and NCUG-SGA groups were significantly higher than those in the AGA group (serum insulin: *P*<0.001, *P* = 0.001; HOMA-IR: *P*<0.001, *P*<0.001; respectively). Moreover, the serum insulin (*P* = 0.006) and HOMA-IR (*P* = 0.002) values in the CUG-SGA were higher than in the NCUG-SGA rats (Table 3).

**Table 3:** Serum insulin levels and HOMA-IR values of CUG-SGA, NCUG-SGA rats and AGA rats.

Variable	AGA(n = 56)	CUG-SGA(n = 54)	NCUG-SGA(n = 69)
**GLU(mmol/L)**			
Males(n = 31,34,and 36)	4.52±0.17	5.37±0.12	4.97±0.08
Females(n = 25,20,and 33)	4.73±0.15	5.58±0.15	4.65±0.09
**INS(mU/ml)**			
Males	10.25±0.33	39.64±1.68*^#^	25.16±1.13*
Females	9.56±0.24	40.87±1.53*^#^	26.07±0.86*
**HOMA-IR**			
Males	2.05±0.29	10.06±0.65*^#^	5.79±0.69*
Females	1.97±0.31	10.85±0.57*^#^	5.41±0.58*

Appropriate for gestational age (AGA), small for gestational age with catch-up growth (CUG-SGA), small for gestational age with no catch-up growth (NCUG-SGA), homoeostasis model assessment for insulin resistance (HOMA-IR). *P<0.05 CUG-SGA or NCUG-SGA *vs* AGA; ^#^ *P*<0.05 CUG-SGA *vs* NCUG-SGA.

### Expression of signaling molecules in muscle tissue

**Figure 1:**
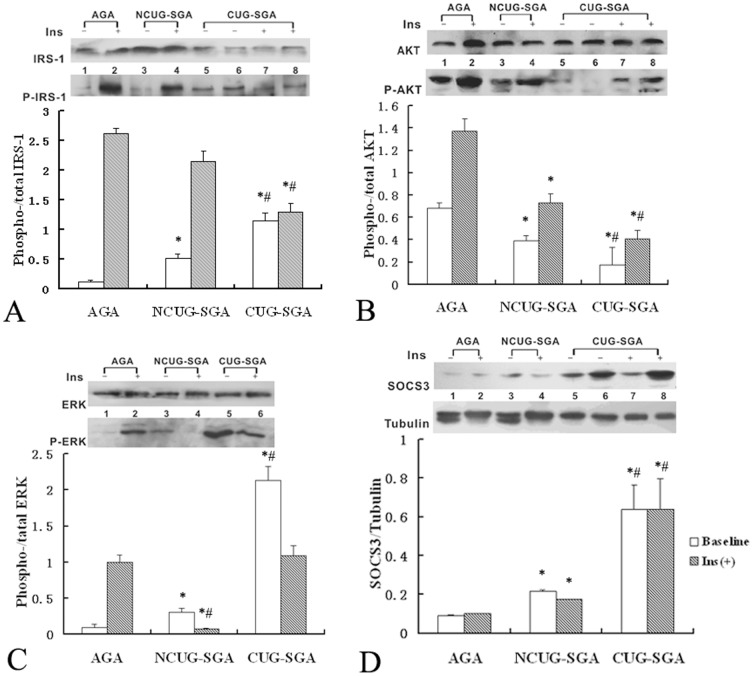
Immunoblot analysis of p-IRS-1, p-AKT, p-ERK and SOCS3 in skeletal muscle cell lysates from CUG-SGA, NCUG-SGA and AGA rats. Rats in appropriate for gestational age (AGA), small for gestational age with catch-up growth (CUG-SGA) and small for gestational age with no catch-up growth (NCUG-SGA) groups (n = 16 for each group) were either intraperitoneal injected intravenously with insulin (Ins) or saline (baseline control) at 4 weeks of age and the skeletal muscles were excised for examination of IRS-1, AKT, ERK, SOCS3 and the phosphorylation of IRS-1, AKT, ERK (p-IRS-1/p-AKT/p-ERK) via Immunoblot analysis. Activation of IRS-1, AKT and ERK were expressed as the ratio of p-IRS-1/p-AKT/p-ERK to total IRS-1, AKT and ERK, respectively. The level of SOCS3 was expressed as the ratio of SOCS3 to tubulin. Data were quantified from 16 samples and were presented as the mean ± SD. *P<0.05 CUG-SGA or NCUG-SGA *vs* AGA; ^#^ *P*<0.05 CUG-SGA *vs* NCUG-SGA.

Immunoblot analysis was performed to test the activation of IRS/PI3K signaling in skeletal muscle tissue 30 min after insulin stimulation. The levels of p-IRS-1 significantly increased after insulin stimulation (*P*<0.05) in the NCUG-SGA and AGA control groups. In contrast, the levels of p-IRS-1 were significantly higher at baseline and did not increase significantly after insulin stimulation in the CUG-SGA group. The levels of p-ERK were also significantly higher at baseline in the CUG-SGA group and decreased to levels comparable to those observed in the AGA group (Figure 1).

The levels of p-AKT after insulin stimulation in the CUG-SGA and NCUG-SGA groups were significantly lower than those in the AGA group. Additionally, the levels of p-AKT in the CUG-SGA group were only 50% of those in the NCUG-SGA group (Figure 1).

Low levels of SOCS3 were observed in AGA control animals under baseline conditions, whereas SOCS3 expression was significantly higher in the CUG-SGA (*P* = 0.001) and NCUG-SGA (*P* = 0.037) rats. Furthermore, the expression of SOCS3 in the CUG-SGA group was significantly higher than that in the NCUG-SGA group (*P* = 0.006). There were no statistically significant differences in the expression of SOCS3 within any group after insulin stimulation (p>0.05).

### Expression of signaling molecules in the skeletal muscle of CUG-SGA rats after *GHR* inhibition

**Figure 2:**
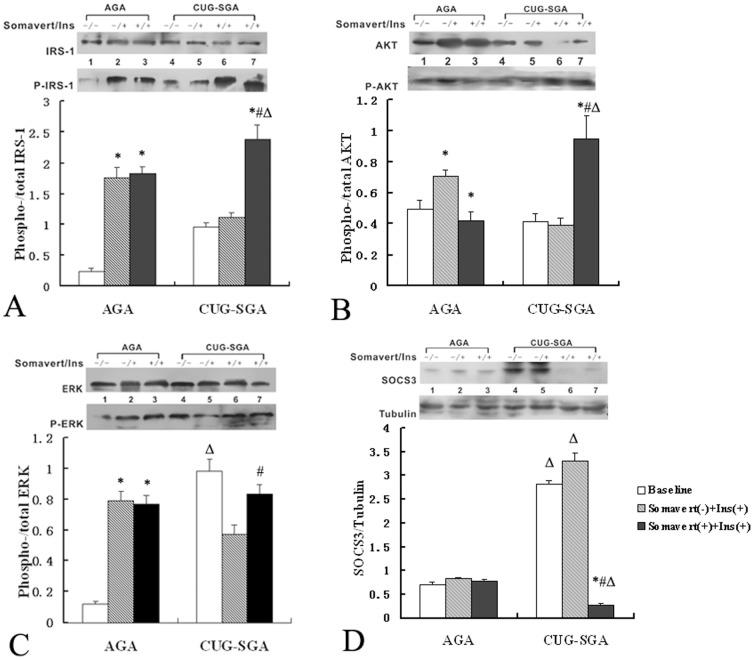
Immunoblot analysis of p-IRS-1, p-AKT, p-ERK and SOCS3 in skeletal muscle cell lysates from CUG-SGA and AGA rats before and after GHR inhibition. Rats in the appropriate for gestational age (AGA) and small for gestational age with catch-up growth (CUG-SGA) groups (n = 16 per group) were further separated into three sub-groups. At four weeks of age, sub-group one received intraperitoneal injection of saline (control), sub-group two received insulin (Ins) stimulation and sub-group three received the GHR inhibitor Somavert before insulin stimulation. The skeletal muscle was then excised for examination of IRS-1, AKT, ERK, SOCS3, and the phosphorylation of IRS-1, AKT, ERK (p-IRS-1/p-AKT/p-ERK) via Immunoblot analysis. Activation of IRS-1, AKT and ERK were expressed as the ratio of p-IRS-1/p-AKT/p-ERK to total IRS-1, AKT and ERK, respectively. The level of SOCS3 was expressed as the ratio of SOCS3 to tubulin. Data were quantified from 16 samples and presented as the mean ± SD. *P<0.05 Somavert(-)+Ins(+) or Somavert(+)+Ins(+) *vs* baseline; ^#^ *P*<0.05 Somavert(-)+Ins(+) *vs* Somavert(+) +Ins(+); Δ*P*<0.05 CUG-SGA *vs* AGA.

As shown in Figure 2, the level of SOCS3 did not significantly change after *GHR* inhibition in the AGA group. However, the expression of SOCS3 in CUG-SGA rats was significantly down-regulated after *GHR* inhibition (*P*<0.001). Likewise, no significant differences were detected in the expression of p-IRS-1 and p-AKT after insulin stimulation in the AGA group after *GHR* inhibition, whereas p-IRS-1, p-AKT and p-ERK were all dramatically up-regulated in the CUG-SGA group after *GHR* inhibition (*P* = 0.001). The reduced levels of p-ERK after insulin stimulation alone were restored to basal levels in the CUG-SGA rats treated with the *GHR* inhibitor.

## Discussion

**Figure 3:**
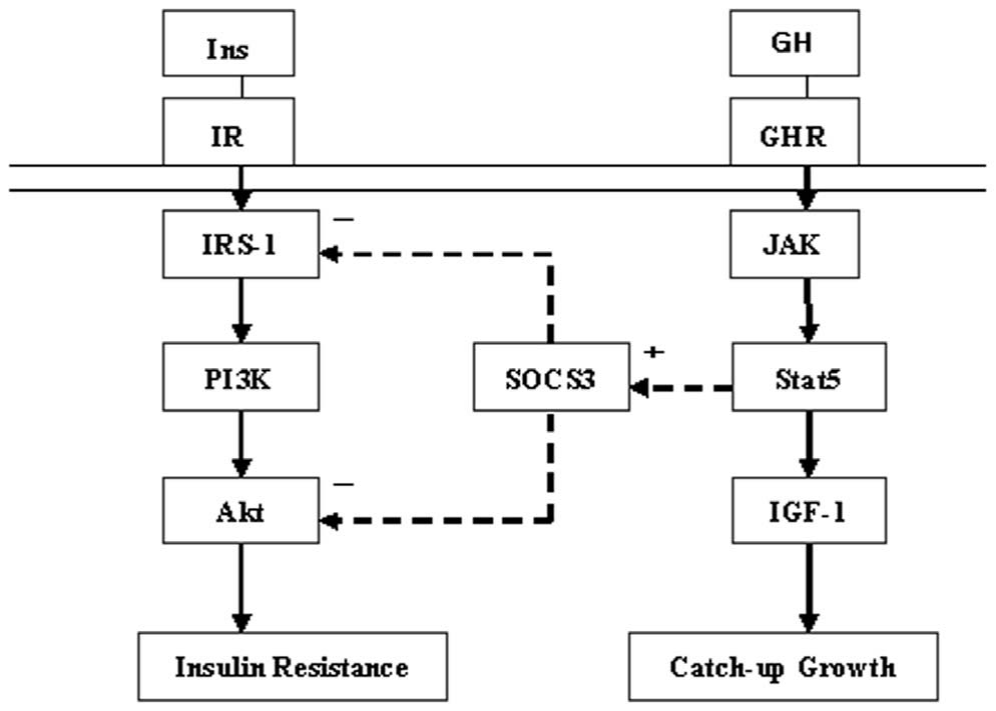
Crosstalk between insulin and GH signaling. GH signaling induces up-regulation of SOCS3 in SGA with Catch-up Growth. Higher SOCS3 levels result in impairment of IRS-1-PI3K-AKT signaling and it may have a role in insulin resistance.

In the present study, we demonstrate that 1) insulin resistance was observed in SGA rats with or without CUG, and rats with CUG show even greater insulin resistance; 2) insulin-stimulated IRS-1 and AKT phosphorylation was significantly blunted in CUG-SGA rats; 3) SOCS3 expression was upregulated in CUG-SGA rats; 4) *GHR* inhibition significantly suppressed SOCS3 but elevated the expression of p-IRS-1, p-AKT and p-ERK in insulin-stimulated CUG-SGA animals. This evidence suggests that insulin resistance in CUG-SGA rats is associated with impairment of IRS-1-PI3K-AKT signaling, which results from *GHR* signaling-induced upregulation of SOCS3 expression (Figure 3).

We found that insulin resistance indexes were much higher in the CUG-SGA group than in the NCUG-SGA and AGA groups. This indicates that SGA rats with CUG demonstrate more significant insulin resistance, which is in accordance with our previous clinical results [6]. Skeletal muscle was the most common and earliest affected tissue during CUG in individuals with SGA [13]. Hence, we next investigated the alteration of molecular pathways in the skeletal muscle of SGA rats with CUG.

IRS-1, a crucial upstream signaling molecule in the insulin signaling pathway, plays a critical role in skeletal muscle cells. Shirakami *et al*. found that knockout of the IRS-1 gene in mice induced profound insulin resistance, insulin-like growth factor resistance, and impaired glucose intolerance after intraperitoneal injection of glucose [14]. De Blasio *et al*. used placental restriction to establish the intrauterine growth restriction sheep model, and showed that placental restriction could decrease the expression of IRS-1 to 28% and AKT to 44% in skeletal muscle. In addition, this positively correlated with insulin resistance without altering insulin levels or related genes expressed in the liver [15]. In the present study, insulin-stimulated IRS-1 and AKT phosphorylation in skeletal muscle were significantly blunted in CUG-SGA rats, which is consistent with a previous report [16], and suggests that impairment of the IRS-1-PI3K-AKT signaling pathway may contribute to the insulin resistance observed in the CUG-SGA rats.

The p-ERK levels in the NCUG- and CUG-SGA groups were significantly increased (chronically stimulated) by 3-fold and 20-fold, respectively. Interestingly, the p-ERK level at baseline and its reaction to insulin stimulation in the CUG-SGA rats was the opposite of what was observed with p-AKT expression in these same animals. Thus, to a certain extent, insulin resistance accompanied with rapid CUG can be demonstrated in infancy. The impairment of the IRS-1-PI3K pathway, occurred in the subjects of CUG-SGA. The IRS-1-ERK pathway may be chronically activated under baseline conditions to promote CUG during early postnatal life in SGA children.

Additionally, no significant difference was found in the expression of p-IRS-1 after *GHR* inhibition in the AGA group, indicating that for AGA, IRS-1 may not respond to GH signaling. However, the levels of p-IRS-1 increased in response to insulin stimulation after inhibition of *GHR* in CUG-SGA group, indicating that abnormal GH signaling may contribute to the inhibition of insulin-IRS-1 signal transduction. Examination of AKT, the downstream signal of IRS-1, demonstrated that the levels of p-AKT decreased after *GHR* inhibition in the AGA group. These findings suggest that GH signaling could induce phosphorylation of AKT in AGA animals under physiological conditions. GH may regulate glucose metabolism, first by its insulin-like effect in acute phase, followed by its antagonistic effect [8]. The expression of p-AKT did not increase when IRS-1 was chronically activated in CUG-SGA, even upon insulin stimulation. The expression of p-AKT increased profoundly after *GHR* inhibition, suggesting that GH signaling may impede the signal transduction of the IRS-1-AKT pathway. Thus, we conclude that GH can affect IRS-1-AKT signal transduction in SGA with CUG. A previous study demonstrated that silencing of the *GHR* gene could ameliorate insulin sensitivity in mice fed high fat diets, decrease the content of triglyceride in muscle and liver, and increase IL-15 levels [17]. Our findings further confirm that GH signaling may impair the metabolic axis.

We investigated another signal pathway, MAPK-ERK, and did not find profound differences in the expression of p-ERK upon insulin stimulation before or after GH inhibition in the AGA group. These results demonstrate that ERK is activated mainly by insulin stimulation in AGA animals under physiological conditions, but not GH signaling. These observations are in accordance with a previous study which indicated that GH signaling could not activate the ERK pathway [18]. The activity of ERK in response to *GHR* inhibition in the CUG-SGA group was similar to that of p-IRS-1, implying the IRS-1-ERK axis may be only slightly influenced or not influenced by GH signaling. In summary, two signaling pathways were affected by *GHR* inhibition: 1) GH signaling may influence the signal transduction of IRS-1-AKT and 2) the MAPK-ERK pathway may be activated to promote growth by chronic insulin stimulation.

SOCS3, described as a suppressor of cytokine signaling, can be activated through the JAK-STAT pathway by GH, insulin or cytokines [19], [20]. SOCS3 competitively inhibits the activation of signaling proteins and is degraded by ubiquitin-mediated proteasomal degradation *in vitro*, thus negatively regulating the insulin signaling pathway [21]. Therefore, high level of SOCS3 expression is closely associated with insulin resistance [22]. In our study, the expression of SOCS3 was significantly increased under baseline conditions in the CUG-SGA and NCUG-SGA groups. SOCS3 expression was greatly reduced after *GHR* inhibition in the CUG-SGA group, and this was accompanied by enhanced activation of IRS-1 and AKT. This demonstrates that GH may inhibit the IRS-PI3K-AKT signaling pathway through up-regulation of SOCS3. In SOCS3-silenced hepatocytes, the expression of IRS-1 and PI3K were both elevated, suggesting an important role for SOCS3 in regulating the IRS-PI3K-AKT pathway [23]. The mice of SOCS3 specifically deleted (SOCS MKO) showed enhanced skeletal muscle insulin receptor substrate 1 (IRS-1) and AKT phosphorylation [24]. Our present study further confirms this hypothesis using *in vivo* tissue samples.

In conclusion, our current study demonstrates that SGA rats with CUG exhibit increased insulin resistance. Moreover, insulin resistance in CUG-SGA rats is associated with impairment of the IRS-1-PI3K-AKT signaling pathway, which results from GH signaling-induced upregulation of SOCS3 expression.
